# A circular RNA derived from the ryanodine receptor 2 locus controls cardiac hypertrophy and calcium handling

**DOI:** 10.1007/s00018-025-05915-2

**Published:** 2025-10-21

**Authors:** Wen Pan, Hannah J. Hunkler, Shambhabi Chatterjee, Dongchao Lu, Isabelle Riedel, Anika Gietz, Ke Xiao, Maximilian Fuchs, Dimyana Neufeldt, Theresia Kraft, Cheng-Kai Huang, Sarah Cushman, Anne Bührke, Arne Schmidt, Elisa Mohr, Natalie Weber, Christian Bär, Thomas Thum

**Affiliations:** 1 Institute of Molecular and Translational Therapeutic Strategies, Hannover Medical School, Carl-Neuberg-Str. 1, 30625 Hannover, Germany; 2 Center for Translational Regenerative Medicine, Hannover Medical School, Hannover, Germany; 3 Fraunhofer Institute for Toxicology and Experimental Medicine, Hannover, Germany; 4 Fraunhofer Cluster of Excellence Immune-Mediated Diseases (CIMD), Hannover, Germany; 5 School of Integrative Medicine, Shanghai University of Traditional Chinese Medicine, Shanghai, China; 6 Institute for Molecular and Cell Physiology, Hannover Medical School, Hannover, Germany; 7 Dean’s Office for Academic Career Development, nextGENERATION Medical Scientist Program, Hannover Medical School, Hannover, Germany

**Keywords:** Cardiac hypertrophy, Calcium handling, Non-coding RNA, Circular RNA, Heart failure, Ryanodine receptor

## Abstract

Heart failure (HF) is a leading cause of mortality worldwide, characterized by structural and functional alterations that result in reduced cardiac function. During the progression of HF, cardiomyocytes undergo profound remodeling – including development of pathological hypertrophy and impaired calcium handling – which exacerbates cardiac dysfunction. Given the limited efficacy of current pharmacological treatments, there is an urgent need for novel mechanistically-orientated therapeutic strategies. Circular RNAs (circRNAs), a recently identified sub-class of non-coding RNAs, have emerged as pivotal regulators of diverse cellular processes in homeostasis and disease. We identified a significant repression of circRYR2 (mmu_circ_0000431), derived from the ryanodine receptor 2 (RYR2) locus, through global RNA profiling in hypertrophic compared to healthy murine hearts. Notably, a decreased expression of the human circRYR2 homologue hsa_circ_0112647 was also found in human failing hearts. Loss-of-function experiments in murine and human cardiomyocytes resulted in hypertrophic responses, whereas AAV-based overexpression of circRYR2 prevented cytokine-induced cardiomyocyte hypertrophy. Whole transcriptome analysis after circRYR2 depletion identified massive perturbations of calcium handling and contractility pathways. Indeed, modulation of circRYR2 affected the expression of the sarco/endoplasmic reticulum Ca²⁺ ATPase 2a (SERCA2a), and consequently, Ca^2+^ transients were assessed, revealing impaired or improved Ca^2+^ handling after circRYR2 knockdown or overexpression, respectively. In summary, the findings presented here provide new mechanistic insights for the circular RNA transcript derived from RYR2 locus and support circRYR2 overexpression strategies as promising therapeutic strategies for cardiac dysfunction.

**Graphical abstract:**

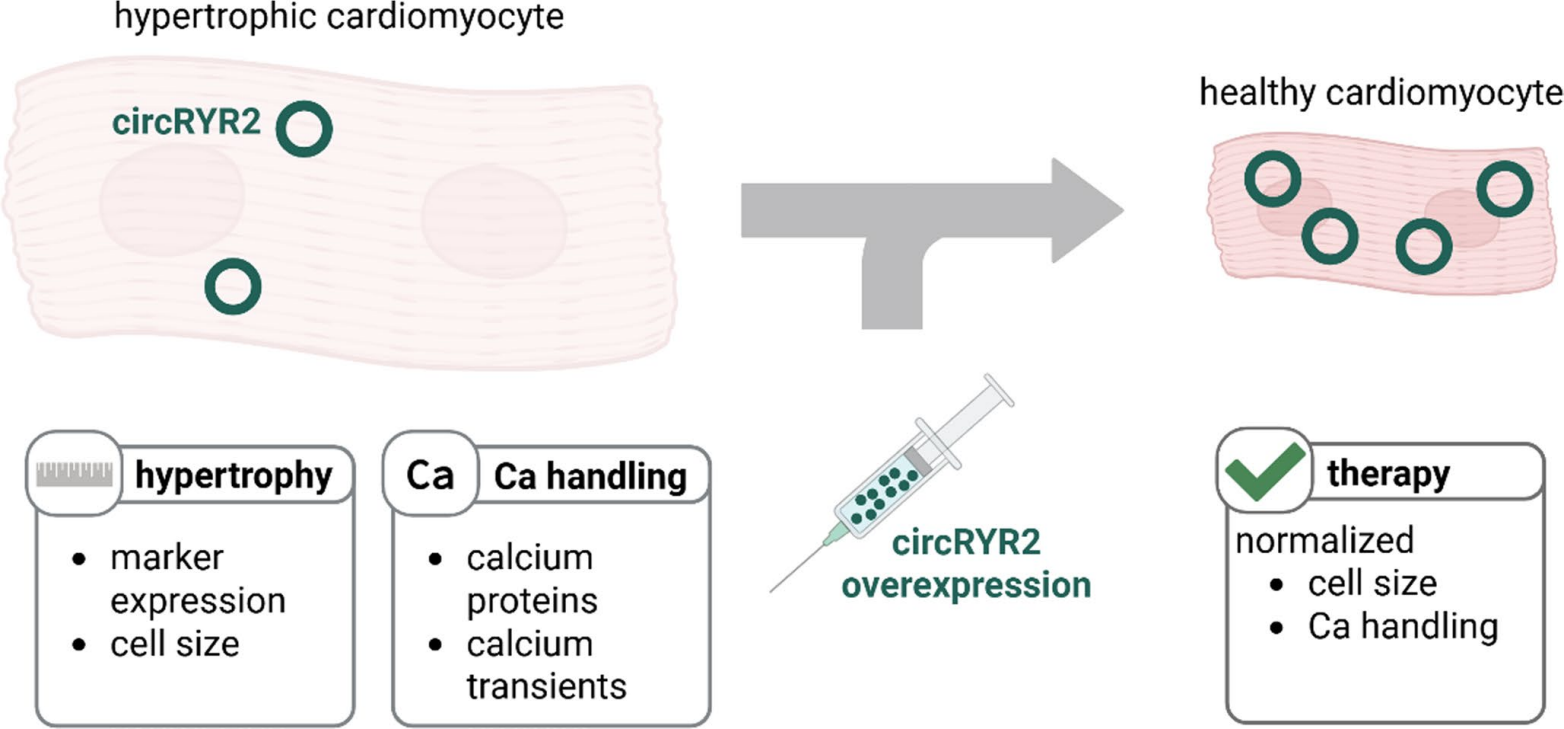

**Supplementary Information:**

The online version contains supplementary material available at 10.1007/s00018-025-05915-2.

## Introduction

In failing hearts, cardiomyocytes exhibit numerous pathological alterations that contribute to the clinical syndrome of heart failure (HF). These include changes in cardiomyocyte survival, hypertrophy, metabolism and contractile function. HF remains a leading cause of mortality worldwide, imposing a substantial socioeconomic burden [1]. Despite the diverse and growing patient population, treatment options remain limited, with therapeutic efforts focusing on symptom management and life extension rather than disease resolution. Pharmacological treatments for HF patients are still limited, highlighting an urgent need for alternative therapeutic strategies.

One promising avenue involves targeting non-coding RNAs (ncRNAs), a molecule class that possess regulatory functions without encoding proteins themselves [2–6]. Indeed, the first-in-class microRNA (miRNA) inhibitor is currently undergoing clinical trials for HF treatment [7, 8], while a number of other ncRNAs show considerable potential as future pharmacological interventions in pre-clinical studies [9, 10]. Among these, circular RNAs (circRNAs) – a specific subclass of ncRNAs – stand out due to their unique covalently closed loop structure, which confers enhanced stability compared to linear RNAs [6, 11]. CircRNAs are produced during pre-mRNA processing through a non-canonical splicing mechanism known as back-splicing. They are often highly conserved between species and demonstrate tissue-, cell type-, or condition-specific expression patterns [11]. Functionally, circRNAs can act as miRNA sponges (competing with endogenous RNAs), bind proteins as a decoy or scaffold, or even serve as templates for micro-peptides [11]. While there are several examples for disease-associated circRNAs, the molecular mechanisms underlying the functions of many circRNAs remain elusive to date and are under active investigation.

Nevertheless, recent advances in ncRNA research have highlighted their pivotal roles in regulating cellular functions, including processes critical for cardiac health and disease. Among these, calcium (Ca²⁺) handling is particularly essential for maintaining the rhythmic contractions of the heart and ensuring the supply of oxygen and nutrients to the body’s organs. The synchronous contraction of the heart muscle relies on the precise transduction of electrical signals into mechanical action via temporal alterations in cytoplasmic Ca²⁺ concentrations. On the cardiomyocyte level, membrane depolarization leads to an initial Ca^2+^ influx through the L-type voltage-gated Ca^2+^ channels triggering a subsequent release of Ca^2+^ through the ryanodine receptor 2 (RYR2) from the sarcoplasmic reticulum (SR) resulting in a contraction. Sarco/endoplasmic reticulum Ca²⁺-ATPase 2a (SERCA2a) pumps Ca^2+^ back into the SR leading to the relaxation of the muscle cells. Excitation-contraction coupling is facilitated and further modulated by numerous other proteins and multiple regulatory layers to ensure rhythmic contractions. However, in diseased hearts, this process is profoundly disrupted [12]. Hypertrophic stimuli promote aberrant Ca²⁺ release decoupling electrical signals from Ca²⁺ dynamics [13]. These disruptions lead to transcriptional changes, which activate pro-hypertrophic gene programs. Structural remodeling in the diseased heart, including alterations in the T-tubular network, further impairs RYR2 localization and activation. Notably, the intertwined processes of hypertrophy and defective Ca²⁺ handling present compelling targets for therapeutic intervention.

In this study, we build on the emerging significance of ncRNAs, particularly circRNAs, in cardiac biology. Through circRNA profiling in hypertrophic hearts, we identified circRYR2, a circular RNA derived from the RYR2 locus. Functional experiments involving loss- and gain-of-function approaches revealed circRYR2 as a crucial regulator of both cardiac hypertrophy and Ca²⁺ handling, positioning it as a promising target for novel therapeutic strategies.

## Results

### Identification of the conserved circrna circRYR2 in cardiac remodeling

**Fig. 1:**
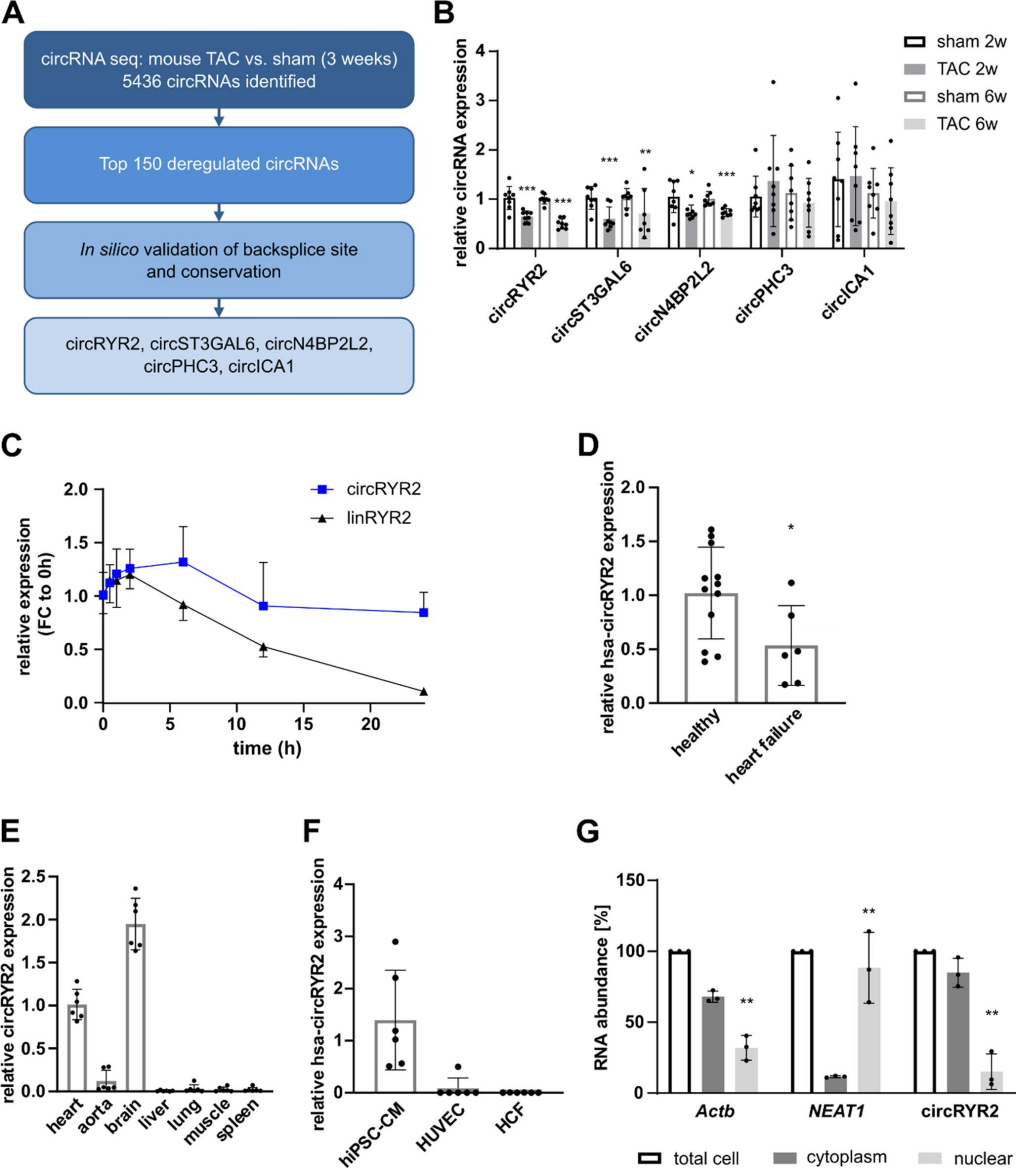
Identification of circRYR2 in hypertrophic mouse hearts **A** Filtering strategy after circRNA profiling in hypertrophic mouse hearts 3 weeks after transverse aortic constriction (TAC). **B** Expression levels of top five regulated circRNAs in murine cardiac tissue 2 and 6 weeks after TAC (*n* = 8). **C** Expression levels after actinomycin D treatment in HL-1 cells (*n* = 6). **D** Hsa-circRYR2 expression in human failing hearts (*n* = 6–12). **E** CircRYR2 expression in murine organs (*n* = 6). **F** Hsa-circRYR2 expression in main cell types of the heart: cardiomyocytes (human iPSC-CMs), endothelial cells (HUVEC), cardiac fibroblasts (HCF) (*n* = 6). **G** Expression levels after subcellular fractionation in HL-1 cells (*n* = 3). *P* values were calculated using two-tailed Student’s *t*-test

To study potential changes of circRNAs during cardiac hypertrophy and remodeling, we induced left ventricular pressure overload by transverse aortic constriction (TAC) in mice. Global circRNA profiling of the ventricular tissue was performed three weeks after the surgery. 5436 circRNAs were identified, of which only differentially expressed circRNAs were considered for further analyses (fold change > 1.5) (Fig. 1A, Supplementary Fig. 1A). CircRNAs with a low sequence conservation between rodents and humans were also disregarded. Of the remaining circRNAs, the regulation of the five top candidates circRYR2 (mmu_circ_0000431, hsa_circ_0112647), circST3GAL6 (mmu_circ_0006351, hsa_circ_0066608), circN4BP2L2 (mmu_circ_0012369, hsa_circ_0000471), circPHC3 (mmu_circ_0010619, hsa_circ_0001360), and circICA1 (mmu_circ_0013575, hsa_circ_0079422) were next validated by qRT-PCR in independent samples from pressure-overloaded mouse hearts two and six weeks after TAC or sham surgery (Fig. 1B). CircRYR2, circST3GAL6, and circN4BP2L2 were significantly downregulated in hypertrophic hearts, while the expression of circPHC3 and circICA1 showed highly variable expression two and six weeks after the TAC surgery. For further functional analysis, we chose circRYR2 as a promising candidate based on the prominent role of its host gene, RYR2, in cardiac function and disease. From this locus, five other circRNAs are annotated, however they were either not detectable or not differentially expressed in the sequencing data set (Supplementary Fig. 1B). Strikingly, the host gene expression remained unaltered during cardiac remodeling (Supplementary Fig. 1C). RYR2 is mainly expressed in cardiomyocytes with crucial function in intracellular Ca^2+^ release [14]. We thus postulated that the circRNA originating from this gene may also play a significant role in cardiomyocyte function. The murine circRYR2 is formed by the back-splicing of the 3’-end of exon 29 to the 5’-end of exon 24 of RYR2. The circular nature of this transcript was validated by PCR amplification with divergent primers and subsequent Sanger sequencing confirmed the backsplicing of exon 29 to 24 (Supplementary Fig. 1D, E). To further validate the closed loop structure of the transcript, the heightened stability of the circular compared to linear configuration was demonstrated by increased abundance of circRYR2 after transcriptional inhibition by actinomycin D and resistance towards exonuclease RNase R treatment (Fig. 1C, Supplementary Fig. 1 F). Importantly, the human homologue of circRYR2 (hsa-circRYR2) was downregulated in failing human hearts compared to healthy cardiac tissue, in line with the regulation in TAC-induced hypertrophic murine hearts, strengthening its translational potential (Fig. 1D). CircRYR2 was highly expressed both in the brain and the heart (Fig. 1E). Among the most abundant cell types of the heart, the murine and human circRYR2 was predominantly present in cardiomyocytes, which suggest an analogous distribution to that of the host gene (Fig. 1F, Supplementary Fig. 1G). Within cardiomyocytes, circRYR2 was mainly localized in the cytoplasm (Fig. 1G).

Collectively, we validated circRYR2 as a bona fide circRNA. Its expression and biodistribution profiles suggest it as a circRNA with regulatory potential in cardiac remodeling in both mice and humans.

### Loss of circRYR2 promotes cardiomyocyte hypertrophy

**Fig. 2:**
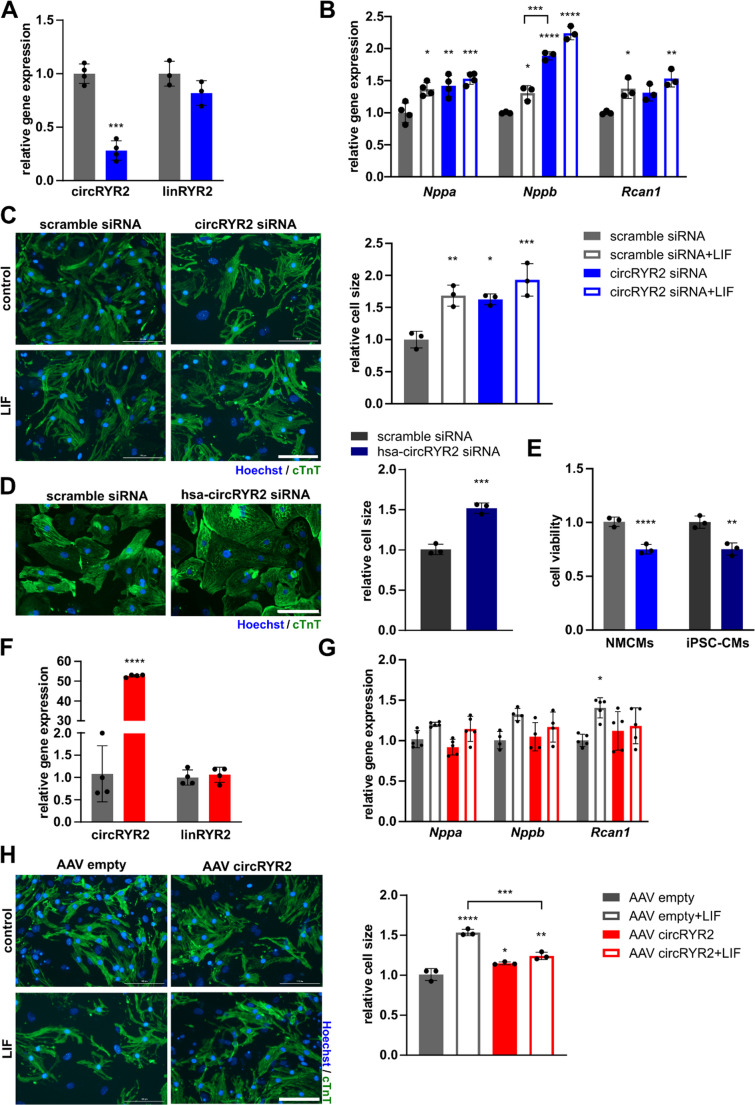
CircRYR2 regulates cardiomyocyte hypertrophy and cell viability **A** Expression levels after circRYR2 knockdown in NMCMs (*n* = 3–4). **B** Expression levels of hypertrophic genes after siRNA-mediated circRYR2 knockdown and hypertrophic stimulation with leukemia induced factor (LIF) (*n* = 3–4). **C** Cell size assessment in NMCMs after circRYR2 knockdown and LIF stimulation (*n* = 3). Scale bar 100 μm. **D** Cell size assessment in human iPSC-CMs (*n* = 3). Scale bar 100 μm. **E** Cell viability after circRYR2 knockdown in NMCMs and human iPSC-CMs (*n* = 3). **F** Expression levels after circRYR2 overexpression in NMCMs (*n* = 4). **G** Expression levels of hypertrophic genes after circRYR2 overexpression and hypertrophic stimulation with LIF (*n* = 6). **H** Cell size assessment in NMCMs after circRYR2 overexpression and LIF stimulation (*n* = 3). Scale bar 100 μm. *P* values were calculated using two-tailed Student’s *t*-test or one-way ANOVA with Bonferroni correction for more than two conditions

To induce a hypertrophic phenotype in vitro, neonatal mouse cardiomyocytes (NMCMs) were stimulated with different hypertrophic stimuli, which resulted in reduced circRYR2 abundance (Supplementary Fig. 2 A). For the characterization of circRYR2, eccentric hypertrophy was induced by leukemia inhibitory factor (LIF) [15]. To investigate the functional role of circRYR2 in cardiac (patho-) biology, siRNAs targeting the backsplice site were designed, allowing specific knockdown of the circRNA without influencing the host gene expression (Fig. 2A). CircRYR2 silencing induced a hypertrophic response indicated by upregulation of the hypertrophic genes *Nppa*, *Nppb* and *Rcan1* comparable or even stronger than with the hypertrophic LIF treatment alone (Fig. 2B). Accordingly, cardiomyocyte size increased significantly after knockdown of circRYR2, to a similar extent as pro-hypertrophic LIF treatment (Fig. 2C). In line with the knockdown in murine cardiomyocytes, siRNAs were designed targeting the human circRYR2 (hsa-circRYR2) backsplice site, which also induced cellular hypertrophy in human induced pluripotent stem cell-derived cardiomyocytes (iPSC-CMs) (Fig. 2D, Supplementary Fig. 2B). Silencing of circRYR2 also reduced the viability of murine and human cardiomyocytes (Fig. 2E), suggesting that circRYR2 might play a role in overall cardiomyocyte health, while the oxygen consumption rate was not altered in rodent cardiomyocytes (Supplementary Fig. 2C, D).

### CircRYR2 overexpression is protective after hypertrophic stimulation

We next tested overexpressing circRYR2 as a potential therapeutic strategy to prevent hypertrophy. To do so, a construct with the species-specific circRYR2 sequence and ALU elements (as inverted repeats up- and downstream of the circRYR2 exon sequences to facilitate the back-splicing), was designed and an AAV6-mediated delivery was chosen (Supplementary Fig. 2E). The transduction of NMCMs with AAV particles led to a significant overexpression of circRYR2 without influencing the expression of the host gene RYR2 (Fig. 2F). The overexpression of the correct circRNA sequence was validated by gel electrophoresis and Sanger sequencing (Supplementary Fig. 2F). Same construct was designed and tested to overexpress the human homologue of circRYR2 (Supplementary Fig. 2G-H). The overexpression of circRYR2 in NMCMs under basal condition did not alter hypertrophic gene expression, however in hypertrophic NMCMs the expression of *Rcan1* was reduced (Fig. 2G). Overexpression of circRYR2 in LIF-stimulated cardiomyocytes prevented cardiomyocyte hypertrophy (Fig. 2H).

As hypothesized from the circRNA profiling, circRYR2 emerged as a regulator of cardiac hypertrophy and its overexpression mitigated the hypertrophic phenotype.

### CircRYR2 regulates calcium handling of cardiomyocytes

**Fig. 3:**
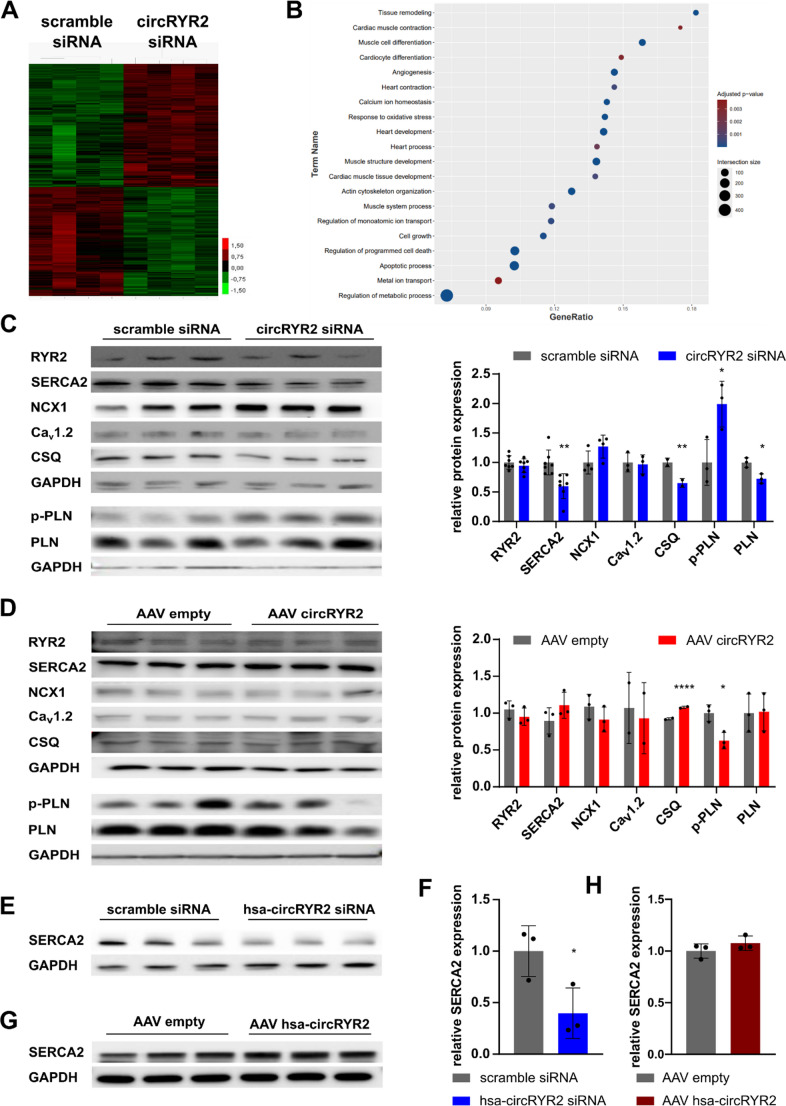
Modulation of circRYR2 impacts Ca^2+^ handling of cardiomyocytes **A** Heatmap of transcriptome analysis after circRYR2 knockdown in NMCMs (*n* = 4). **B** Significantly deregulated key pathways. **C** Protein quantification in HL-1 cells after circRYR2 knockdown by Western blotting (*n* = 2–7). **D** Protein quantification in HL-1 cells after circRYR2 overexpression by Western blotting (*n* = 3–4). **E** Protein quantification in human iPSC-CMs after hsa-circRYR2 knockdown by Western blotting (*n* = 3). **F** Protein quantification in human iPSC-CMs after circRYR2 overexpression by Western blotting (*n* = 3). *P* values were calculated using two-tailed Student’s *t*-test

To elucidate the functional role of circRYR2 further, we performed global transcriptome analysis after circRYR2 silencing (Fig. 3A). 1368 genes were significantly differentially expressed after circRYR2 knockdown (adjusted *P* < 0.05). Gene ontology analysis was performed to identify altered key pathways and biological processes after circRYR2 inhibition (Fig. 3B). Here, loss of circRYR2 expression (as observed in cardiac hypertrophy and pathological remodeling) was mainly associated with pathways of dysregulated Ca^2+^ signaling and contractility. To visualize relevant transcriptional changes, we curated a list of representative Ca²⁺-associated genes based on the top differentially expressed genes within the GO terms connected to Ca^2+^ signaling (Supplementary Fig. 3A). While this panel captures a broad range of affected genes, we note that some canonical Ca²⁺-handling genes (e.g. *ATP2A2*, *CACNA1C*, *CASQ2*, *PLN*) were not included due to not meeting the top DEG cutoff. Nonetheless, individual expression analyses for these classical genes showed a regulation of those in human iPSC-CMs highlighting additionally the translational potential of the findings from mouse to man (Supplementary Fig. 3B, C). In addition to several Ca^2+^- and contractility-associated genes, general hypertrophic and developmental-associated pathways were regulated. This finding prompted us to investigate potential regulatory effects of circRYR2 on proteins relevant for the excitation-contraction coupling in cardiomyocytes. Therefore, protein expression of RYR2, SERCA2, NCX1, Ca_v_1.2, CSQ, phosphorylated and total PLN was analyzed after modulating circRYR2 in murine cardiomyocytes (Fig. 3C, D). While protein expression of the host gene RYR2 was unaltered after circRYR2 knockdown, the protein expression of SERCA2, CSQ and total PLN were significantly reduced, while the amount of phosphorylated PLN was significantly increased. Reduced SERCA2 levels are indicative for slower relaxation and the increased PLN phosphorylation may compensate for reduced SERCA2 levels to preserve relaxation. In line with changes of the murine protein expression levels, also in human iPSC-CMs, *ATP2A2* (codes for SERCA2) and *CASQ2* expression levels were significantly lower after circRYR2 silencing (Supplementary Fig. 3B). SERCA2 was also significantly reduced on protein level in human iPSC-CMs (Fig. 3E), which strengthens the translatability from mouse to human and indicates that circRYR2 might also interfere with the transcription of these Ca^2+^-associated proteins.

**Fig. 4:**
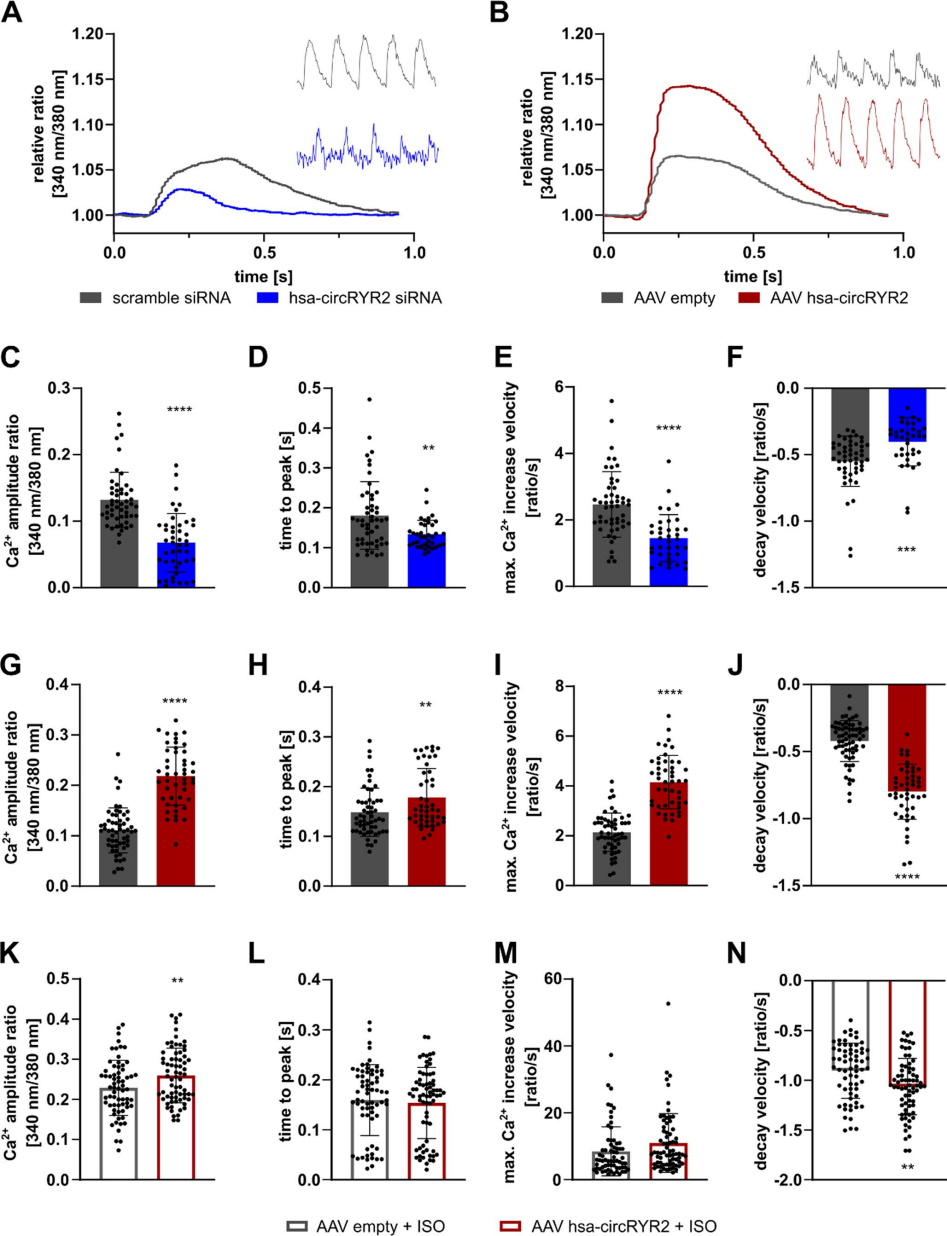
Modulation of circRYR2 influences intracellular Ca^2+^ transients in cardiomyocytes **A** Mean time courses of intracellular Ca^2+^ transients upon electrical stimulation were assessed after hsa-circRYR2 silencing in human iPSC-CMs. Representative recordings of Fura-2 emission over 5 s are depicted on right side. **B** Mean time courses of intracellular Ca^2+^ transients were assessed upon electrical stimulation after hsa-circRYR2 overexpression in human iPSC-CMs. Representative recordings of Fura-2 emission over 5 s are depicted on right side. **C-F** Parameters of Ca^2+^ transients after circRYR2 knockdown (from two independent cardiac differentiations, 2–10 cells per 2–4 coverslip). **G-J** Parameters of Ca^2+^ transients after circRYR2 overexpression (2–10 cells per 2–4 individual coverslips derived from two independent cardiac differentiations). **K-N** Parameters of Ca^2+^ transients after circRYR2 overexpression and acute isoprenaline (ISO, 1 µM) stimulation (from two independent cardiac differentiations, 7–17 cells per 2–4 coverslip each). *P* values were calculated using two-tailed Student’s *t*-test

The overexpression of circRYR2 in murine cardiomyocytes left RYR2 abundance unaltered (Fig. 3D), led to a moderate increase of SERCA2 (*P* = 0.214) and significantly increased CSQ expression. The phosphorylation of PLN was reciprocally regulated as seen after circRYR2 silencing. The overexpression in human iPSC-CMs only showed an upregulation of *CACNA1C* (coding for Ca_v_1.2), which gave highly variable results at the protein level (Supplementary Fig. 3C). Also, in human iPSC-CMs SERCA2 showed a slight increase, but less robust as in murine cardiomyocytes (Fig. 3F). Overall, the overexpression at basal condition had less impact on the here studied Ca^2+^-associated genes in murine and human cardiomyocytes compared to the silencing of circRYR2. These findings suggest that the modulation of circRYR2 impacts the abundance of specific Ca^2+^-associated proteins, which might influence Ca^2+^ homeostasis and therefore contractility of cardiomyocytes. To investigate whether those changes are also mirrored in altered Ca^2+^ handling, we measured intracellular Ca^2+^ transients in human iPSC-CMs loaded with the Ca^2+^ indicator Fura-2. Strikingly, both knockdown and overexpression of hsa-circRYR2 altered intracellular Ca²⁺ transients compared to controls (Fig. 4A, B). Knockdown reduced the Fura-2 amplitude, time to peak, and both maximal Ca²⁺ increase and decay velocities (Fig. 4C-F), while overexpression led to reciprocal changes (Fig. 4G-J). Baseline Ca²⁺ levels were elevated in both conditions (Supplementary Fig. 3D-I). To assess adrenergic responsiveness, we stimulated cells with ISO. ISO effected predominantly the Ca²⁺ kinetics, which was already reported before for human PSC-CMs (Supplementary Fig. 3J) [16]. In circRYR2-overexpressing cells, ISO induced only a slight further enhancement of Ca²⁺ amplitude, times and velocities, suggesting reduced adrenergic sensitivity (Fig. 4K-N). These results indicate that circRYR2 modulates Ca²⁺ handling and β-adrenergic responsiveness in a context-dependent manner and may support contractile function under stress.

Taken together, these findings point to a mechanistic role of circRYR2 in the regulation of cardiac hypertrophy and contractility in mouse and human cardiomyocytes.

## Discussion

Although the socioeconomic burden of HF is high and an alarming increase in patient numbers are expected in the upcoming years due to global population ageing, the therapeutic options are still limited [1]. In this regard, ncRNAs have emerged as a new druggable molecule class with the first candidate reaching clinical trials [7, 8].

In this study, we performed circRNA profiling in murine hypertrophic hearts, identifying various differentially expressed circRNAs, which might play functional roles in the development of hypertrophy and therefore, serve as therapeutic candidates. To enable later clinical translation, only those circRNAs with conserved human sequences were selected. Following extensive in silico and in vitro analysis we selected circRYR2 as also its host gene is relevant for cardiac function.

CircRYR2 exhibits cardiomyocyte-specific expression similar to its host gene RYR2, which is crucial for the Ca^2+^-dependent Ca^2+^ release from the sarcoplasmic reticulum into the cytoplasm leading to cardiomyocyte contraction. Of note, experimental modulation of RYR2 expression has major effects on cardiomyocyte contractility [17], specific mutations of RYR2 in humans result in severe arrhythmic syndromes, which might be lethal [18]. Interestingly, our circRNA profiling and qPCR-based validation in hypertrophic murine and human hearts, and hypertrophic stimulated cardiomyocytes in vitro, revealed a regulation of circRYR2, while the host gene expression remained unchanged. A host gene-independent effect was recently reported for several circRNAs, derived for instance, from the INSR or ZFPM2 locus [5, 6]. Although these circRNAs function in host gene unrelated pathways, circRYR2 seems to play an essential role in calcium handling similar to the biological function of its host gene. In this context, while modulation of circRYR2 expression has no impact on RYR2 mRNA or protein expression, another important ion pump for Ca^2+^, the Ca^2+^-ATPase SERCA2, is influenced as demonstrated by significantly lower SERCA2 protein levels when circRYR2 is inhibited. In HF, SERCA2 expression and activity is reduced resulting in reduced cardiac function. Therefore, SERCA2 has been intensively studied as a therapeutic target in the last decades with beneficial effects in vitro as well as in small and large animal models of HF [19–23] leading to clinical trials testing an AAV1-based gene therapy to reintroduce SERCA2a into failing hearts (NCT00454818) [24]. This CUPID trial improved cardiac function in a small cohort of patient, while the following larger CUPID2 phase 2b trial remained neutral on the heart function of the patients (NCT01643330) [25]. This discrepancy might be due to challenges in vector preparations and the delivery of SERCA2a to the myocardium. The following phase 2 trials AGENT-HF (NCT01966887) and SERCA-LVAD (NCT00534703) were interrupted before complete enrollment when CUPID2 results were reported neutral [26, 27]. Therefore, the regulation and finetuning of SERCA2a via the modulation of a circRNA might be a promising strategy. Besides the here tested delivery of circRYR2 via AAV particles, also in vitro transcribed RNA products and their targeted delivery via nanoparticles is a promising and fast developing strategy for specific RNA overexpression approaches [5, 28].

The abundancy and activity of SERCA2a is essential for cardiomyocyte function and its regulation is highly complex. Indeed, there are different RNA molecules reported which orchestrate SERCA2a function in a complex regulatory network. A prominent example is the lncRNA ZFAS1 which directly binds to SERCA2a and inhibits its function, thus the loss of ZFAS1 resulted in improved function in the murine model of myocardial infarction [29]. Moreover, the micro-peptide DWORF (dwarf open reading frame) encoded by a lncRNA, is a prominent example for another level of SERCA regulation in skeletal muscles [30].

In contrast to lncRNAs, inhibitory strategies for circRNAs are challenging due to the sequence identity between the circRNA and the corresponding mRNA. Only the backsplice junction offers a short circRNA-unique sequence, which requires rigorous testing for efficient and circRNA-specific siRNAs. Here, we successfully designed siRNAs which silenced circRYR2 independent of its host gene. The downregulation of circRYR2 resulted in reduced viability and a pro-hypertrophic phenotype in mouse cardiomyocytes with increased hypertrophic marker gene expression and cell size. Importantly, hypertrophic growth after inhibition of hsa-circRYR2 was replicated in human cardiomyocytes, suggesting translational potential. To reverse this phenotype, we designed an AAV-based strategy to overexpress (hsa-) circRYR2 in murine and human cardiomyocytes. Overexpression of circRYR2 rescued the hypertrophic phenotype indicated by reduced hypertrophic markers and cell size. To gain initial mechanistic insight, transcriptome analysis following circRYR2 knockdown revealed not only pathways associated with hypertrophic remodeling but also those related to Ca²⁺ handling. These transcriptomic findings were supported by Western blot analyses showing dysregulation of key calcium regulatory proteins, including decreased SERCA2 and CSQ levels and altered phosphorylation of SERCAs modulator PLN. However, in the setting of circRYR2 overexpression, SERCA2 expression remained unchanged in iPSC-CMs, suggesting that circRYR2 may influence Ca²⁺ handling through post-translational modifications or localized signaling mechanisms rather than through changes in total protein abundance. For functional validation, we quantified intracellular Ca²⁺ transients in human cardiomyocytes following circRYR2 modulation. We observed opposite changes in several parameters upon circRYR2 knockdown and overexpression. Specifically, silencing circRYR2 led to a reduced amplitude and maximum increase and decay velocities of Ca²⁺ transients, whereas overexpression increased their amplitude and respective velocities. Notably, the reduced amplitude and prolonged duration of Ca²⁺ transients observed upon circRYR2 silencing, align with characteristic changes seen in failing cardiomyocytes, where impaired Ca²⁺ handling contributes to reduced contractility [31]. The positive ionotropic and lusitropic effects of acute ISO treatment were reinforced by circRYR2 overexpression indicating that systolic and diastolic properties of the myocardium might be improved.

Hypertrophic signaling and aberrant Ca^2+^ handling are tightly linked and are known to influence each other. High cytoplasmic Ca^2+^ levels, e.g. caused by impaired SERCA2 function, activate calcineurin which in turn activates the transcriptional effector NFAT (nuclear factor of activated T cells), and subsequently the transcription of pro-hypertrophic genes [32, 33]. How circRYR2 is affecting hypertrophy and Ca^2+^ handling exactly in cardiomyocytes needs further detailed investigations.

Taken together, our study highlights circRYR2 as an important regulatory RNA molecule involved in Ca^2+^ handling and cardiomyocyte function. Nevertheless, our work has some limitations that should be mentioned. Firstly, although we clearly demonstrate detrimental and beneficial effects after circRYR2 silencing and overexpression, respectively, the precise mechanism how circRYR2 is influencing Ca^2+^ handling remains to be unveiled. Secondly, although our study highlights a conserved function of circRYR2 between rodents and humans, the experiments were conducted in vitro in isolated primary rodent cardiomyocytes and human iPSC-CMs, which both do not depict all characteristics of adult cardiomyocytes. Thirdly, the exact molecular mechanism warrants further investigation especially how circRYR2 interacts with SERCA2, its regulator PLN and NCX1 under physiological and pathological conditions. Further experiments are warranted investigating the functional role of circRYR2 in mice and human cardiomyocytes as well as three-dimensional cardiac models to ensure future clinical translatability.

## Materials and methods

Detailed methods are provided online in the Supplementary Information.

### Human tissue sampling

Patients’ cardiac tissue was sampled with the approval of the institutional ethics committee of the Hannover Medical School, Germany complied with the 1964 Declaration of Helsinki and its amendments [34, 35]. Detailed patient information is summarized in the Supplementary Information online (Supplementary Table 1).

### Cell culture, treatments and assays

Neonatal mouse or rat cardiomyocytes (NMCMs, NRCMs) were isolated from mouse or rat pups with the Neonatal Heart Dissociation Kit (Miltenyi Biotec) according to manufacturer’s instructions and subsequently maintained. Cardiomyocyte-like HL-1 cells (RRID: CVCL_0303) and human induced pluripotent stem cells (hiPSCs) (MHHi001-A, RRID: CVCL_QX51) [36] were cultured in respective media described in Supplementary Information in detail. HiPSCs were subjected to cardiac differentiation by Wnt modulation as described previously [2, 37]. HEK-293T cells (RRID: CVCL_0063) were cultured according to standard protocols for AAV productions. For a detailed description of cell treatments, procedures and assays see the Supplementary Information.

### Modulation of circRYR2 expression

To knockdown circRYR2, siRNAs were designed targeting the murine or human backsplice site of the circRNA. 100 nM siRNA was transfected with Lipofectamine 2000 (Life Technologies). For overexpression studies, the murine and human circRNA exon sequences, including circularization elements, were cloned into the AAV MCS 1.3 backbone via EcoRI and NotI or BamHI and BstEII restriction sites. The plasmid was then transfected or AAV particles were produced and cells were transduced with the desired MOI.

### Gene expression analysis

For gene expression analysis, RNA was isolated with QIAzol (Qiagen) as per manufacturer instructions and reverse transcribed with hexamer primers of the Biozym cDNA Synthesis Kit. Quantitative real-time PCR (RT-qPCR) was performed using Absolute Blue qPCR SYBR Green Mix (Life Technologies) using Viia7 Real-Time PCR System (Applied Biosystems).

### Measurement of intracellular Ca^2+^ transients with Fura-2 AM

Intracellular Ca^2+^ transients in single human cardiomyocytes were measured using a dual excitation fluorescence photomultiplier system (IonOptix). Human iPSC-CMs were incubated with 1.5 µM Fura-2 AM (Life Technologies) for 25–35 min and washed twice for 15 min. Coverslips with cells were placed into a perfusion chamber and the cells were stimulated to contract at 15 V and 1 Hz at 37 °C as described previously [2, 38]. Fluorescence emission at 510 nm, following alternating excitation at 340 nm and 380 nm was recorded using IonWizard software 6.5 (IonOptix). Autofluorescence was subtracted using untreated control cardiomyocytes. For acute ISO stimulation, 1 µM ISO was infused to the perfusion chamber.

Figures S3J and 4G/4K were conducted in different experimental settings. S3J used iPSC-CMs under physiological conditions and showed a modest ISO response in AAV-control cells. In contrast, the cells used in Fig. 4G K were more responsive to ISO, likely due to increased maturation. In this context, circRYR2-expressing cells again showed a blunted ISO response, suggesting that circRYR2 reduces adrenergic sensitivity in a context-dependent manner. These differences have now been clarified in the revised figure legend and Results section.

### Statistics

All data were plotted and analyzed with GraphPad Prism. Results are presented as mean ± standard deviation (SD). Statistical significance between two groups was determined using an unpaired two-tailed Student’s *t*-test. For comparisons involving three or more groups, one-way analysis of variance (ANOVA) was conducted with Bonferroni correction. A *P*-value of less than 0.05 (*P* ≤ 0.05) was considered statistically significant. Significance levels are indicated as follows: * for *P* ≤ 0.05, ** for *P* ≤ 0.01, *** for *P* ≤ 0.001, and **** for *P* ≤ 0.0001.

## Supplementary Information

Below is the link to the electronic supplementary material.

Supplementary Material 1 (DOCX 2.06 MB )

Supplementary Material 2 (DOCX 67.1 KB)

## Data Availability

All data will be shared upon reasonable request to the corresponding author.

## Abbreviations

ANOVA: one-way analysis of variance
Ca²⁺: calcium
circRNA: circular RNA
DWORF: dwarf open reading frame
HF: heart failure
iPSC-CMs: induced pluripotent stem cell-derived cardiomyocytes
ISO: isoprenaline
LIF: leukemia inhibitory factor
miRNA: microRNA
ncRNAs: non-coding RNAs
NFAT: nuclear factor of activated T cells
NMCMs: neonatal mouse cardiomyocytes
RT-qPCR: quantitative real-time PCR
RYR2: ryanodine receptor 2
SD: standard deviation
SERCA2a: sarco/endoplasmic reticulum Ca²⁺ ATPase 2a
SR: sarcoplasmic reticulum
TAC: transverse aortic constriction
