# Supplementary material for: A circular RNA derived from the ryanodine receptor 2 locus controls cardiac hypertrophy and calcium handling
Source: Cell Mol Life Sci. 2025 Oct 21;82(1):359. doi: 10.1007/s00018-025-05915-2 (PMC12540953; doi:10.1007/s00018-025-05915-2)
Supplement: Supplementary file 1 — Supplementary Material 1 (DOCX 2.06 MB ) [file 18_2025_5915_MOESM1_ESM.docx]

**Supplementary Information**

**A circular RNA derived from the ryanodine receptor 2 locus controls cardiac hypertrophy and calcium handling**

Wen Pan^1,#^, Hannah J. Hunkler^1,#^, Shambhabi Chatterjee^1,3,4^, Dongchao Lu^1,2,5^, Isabelle Riedel^1,3^, Anika Gietz^1^, Ke Xiao^1^, Maximilian Fuchs^1^, Dimyana Neufeldt^1^, Theresia Kraft^6^, Cheng-Kai Huang^1^, Sarah Cushman^1^, Anne Bührke^1^, Arne Schmidt^1,3^, Elisa Mohr^1,3^, Natalie Weber^1,7^, Christian Bär^1,2,3,4*^, Thomas Thum^1,2,*^

^1^ Institute of Molecular and Translational Therapeutic Strategies, Hannover Medical School, Hannover, Germany

^2^ Center for Translational Regenerative Medicine, Hannover Medical School, Hannover, Germany

^3^ Fraunhofer Institute for Toxicology and Experimental Medicine, Hannover, Germany

^4^ Fraunhofer Cluster of Excellence Immune-Mediated Diseases (CIMD), Hannover, Germany

^5^ School of Integrative Medicine, Shanghai University of Traditional Chinese Medicine, Shanghai, China

^6^ Institute for Molecular and Cell Physiology, Hannover Medical School, Hannover, Germany

^7^ Hannover Medical School, Germany, Dean’s Office for Academic Career Development, nextGENERATION Medical Scientist Program

# Wen Pan and Hannah J. Hunkler contributed equally to this work.

* Co-correspondence:

Christian Bär

Institute of Molecular and Translational Therapeutic Strategies, Hannover Medical School

Carl-Neuberg-Str. 1, 30625 Hannover, Germany

baer.christian@mh-hannover.de

Thomas Thum

Institute of Molecular and Translational Therapeutic Strategies, Hannover Medical School

Carl-Neuberg-Str. 1, 30625 Hannover, Germany

thum.thomas@mh-hannover.de


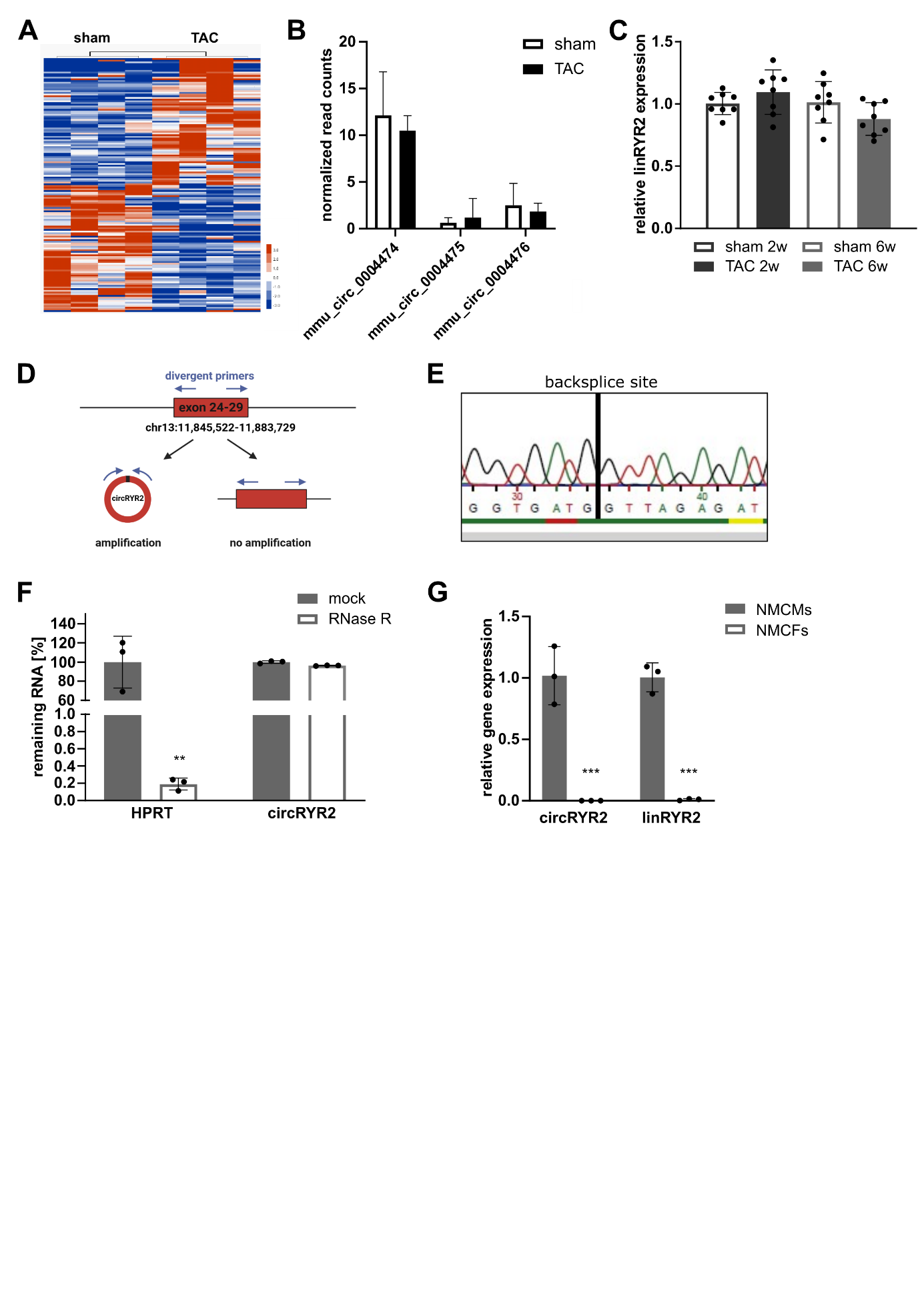
**Supplementary Fig 1** Identification and characterization of circRYR2

**A** Heatmap of circRNA profiling in 3 weeks after transverse aortic constriction (TAC) (n=4). **B** Linear RYR2 expression 2 and 6 weeks after TAC surgery (n=8). **C** Schematics of circRYR2 locus and backsplice site. **D** Sanger sequencing results of circRYR2, backsplice site indicated in black. **E** Relative expression levels after 3 h RNase R treatment of HL-1 cells (n=3). **F** Expression levels in NMCMs and NMCFs (n=3). *P* values were calculated using two-tailed Student’s *t*-test.


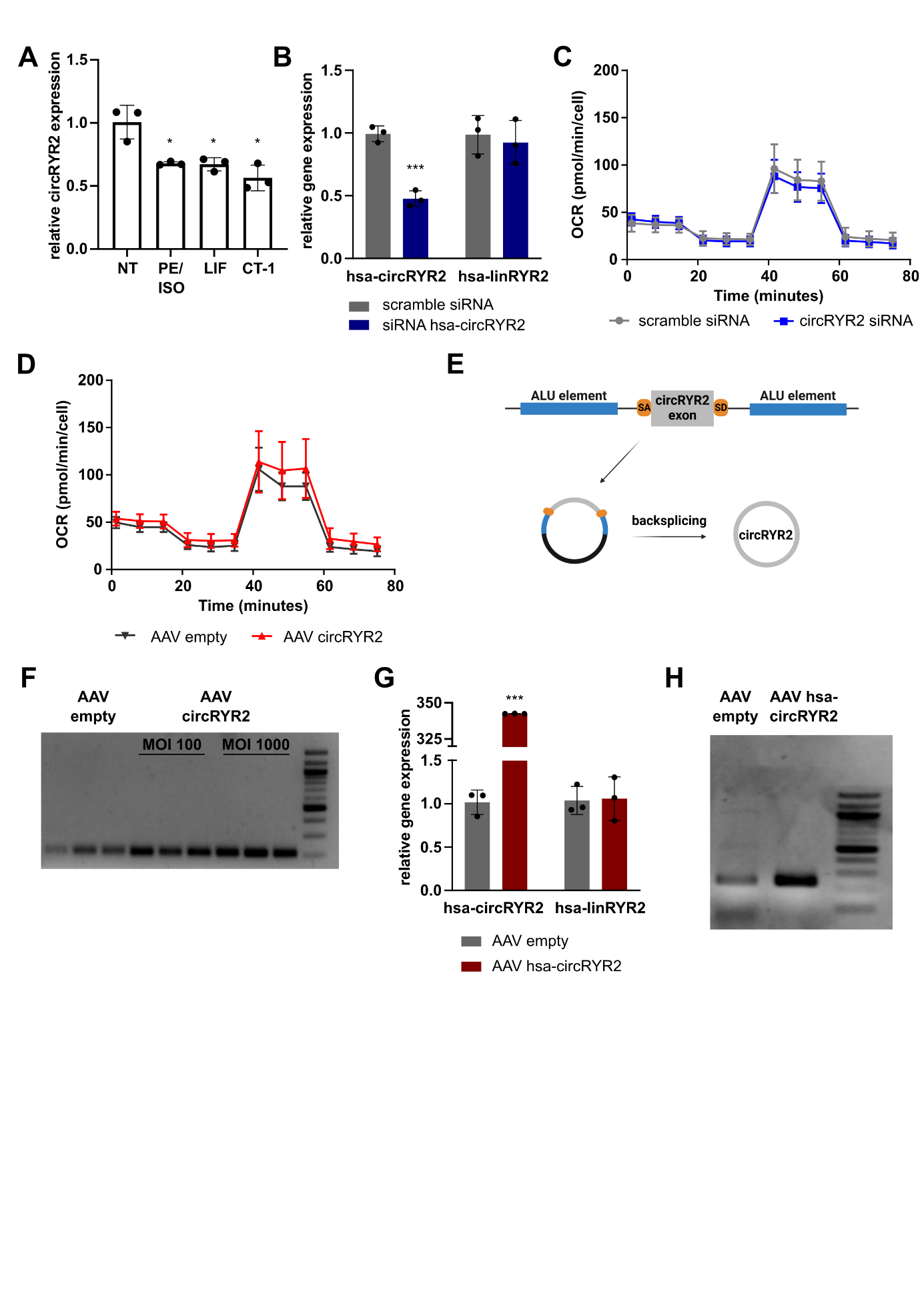


**Supplementary Fig 2** Modulation strategies of circRYR2 by siRNA-mediated knockdown and AAV6-based overexpression

**A** Expression of circRYR2 in HL-1 cells stimulated with 100 µM PE/ISO, 5 nM LIF or 5 nM CT-1 (n=3). **B** Expression levels in human iPSC-CMs after hsa-circRYR2 knockdown (n=3). **C, D** Representative oxygen consumption rate in NRCMs after circRYR2 silencing and overexpression assessed via Seahorse XF Mito Stress test. **E** Schematic representation of overexpression strategy. **F** Gel electrophoresis of RT-qPCR products after circRYR2 overexpression in NMCMs. **G** Expression levels in human iPSC-CMs after hsa-circRYR2 overexpression (n=3). **H** Gel electrophoresis of RT-qPCR products after overexpression in human iPSC-CMs. *P* values were calculated using two-tailed Student’s *t*-test.


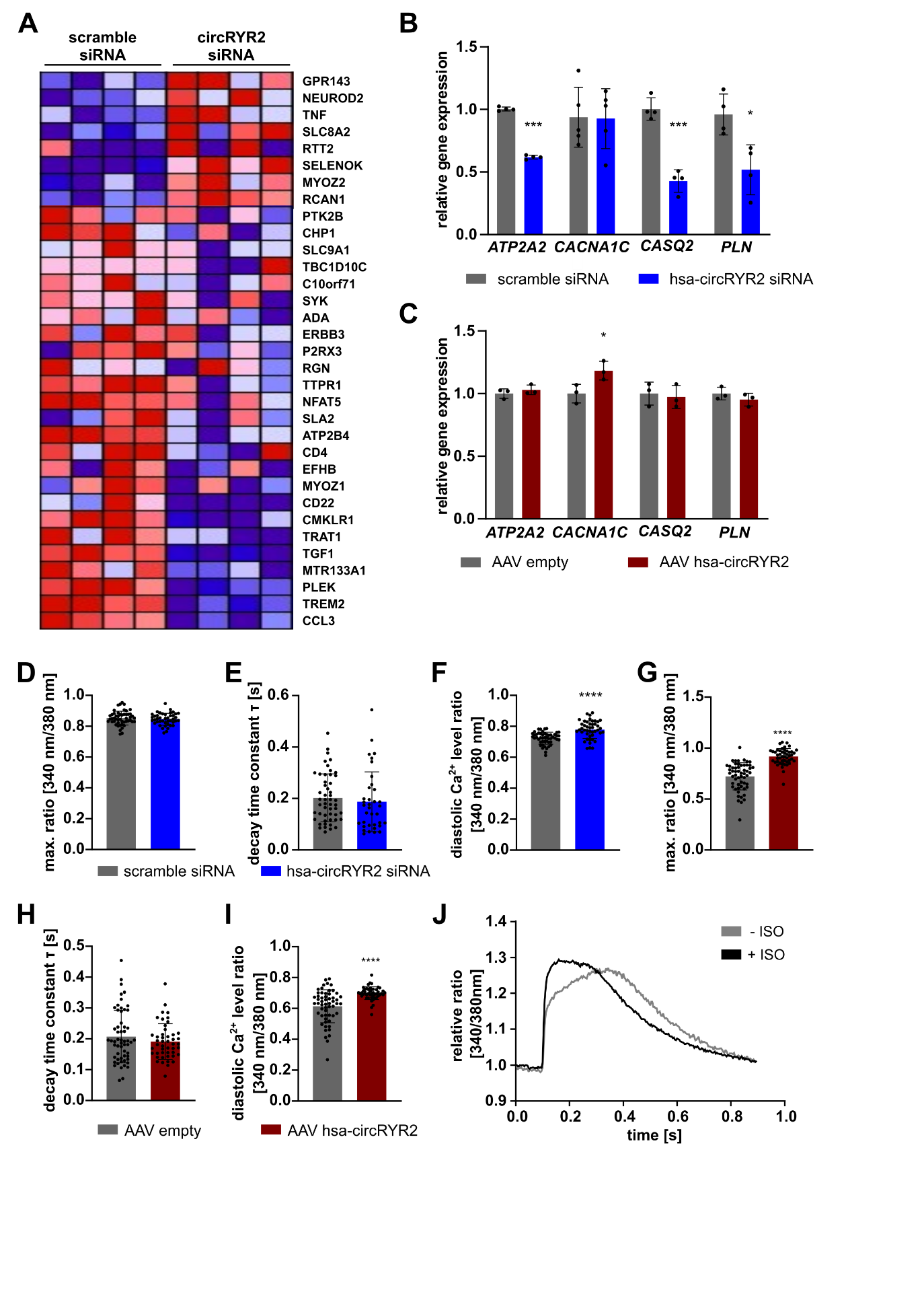


**Supplementary Fig 3** Modulation of circRYR2 influences Ca^2+^ handling

**A** Heatmap showing top DEGs from calcium signaling-related GO terms following circRYR2 knockdown. Genes were selected based on statistical significance (adjusted p-value) and fold change. **B** Expression levels of Ca^2+^-handling genes after hsa-circRYR2 knockdown in human iPSC-CMs (n=4-5). **C** Expression levels of Ca^2+^-handling genes after hsa-circRYR2 overexpression in human iPSC-CMs (n=3). **D-F** Parameters of Ca^2+^ transients assessed in Fura-2 loaded human iPSC-CMs after hsa-circRYR2 knockdown in (2-10 cells per 2-4 individual coverslips derived from two independent cardiac differentiations). **G-I** Parameters of Ca^2+^ transients assessed in Fura-2 loaded human iPSC-CMs after hsa-circRYR2 overexpression (2-10 cells per 2-4 individual coverslips derived from two independent cardiac differentiations). **J** Mean time courses of intracellular Ca^2+^ transients upon electrical stimulation were assessed before and after acute isoprenaline (ISO, 1 µM) stimulation in human iPSC-CMs. *P* values were calculated using two-tailed Student’s *t*-test.
