# Supplementary material for: A circular RNA derived from the ryanodine receptor 2 locus controls cardiac hypertrophy and calcium handling
Source: Cell Mol Life Sci. 2025 Oct 21;82(1):359. doi: 10.1007/s00018-025-05915-2 (PMC12540953; doi:10.1007/s00018-025-05915-2)
Supplement: Supplementary file 2 — Supplementary Material 2 (DOCX 67.1 KB) [file 18_2025_5915_MOESM2_ESM.docx]

#### Supplementary Information

Supplementary Table 1 Summary of human cardiac tissue

|  | total (n) | males (n) | females (n) | mean age (y) ± SD |
| --- | --- | --- | --- | --- |
| control hearts | 12 | 5 | 7 | 45 ± 11 |
| failing hearts | 7 | 4 | 3 | 39 ± 18 |

#### Material and Methods

Animal experiments were approved by the Niedersächsisches Landesamt für Verbraucherschutz und Lebensmittelsicherheit. Male C57BL/6N mice, 6-8 weeks old, underwent either TAC or sham surgery to induce cardiac hypertrophy. Hearts were collected at 2, 3 or 6 weeks post-surgery and RNA was isolated.

##### Cell culture

Neonatal mouse or rat cardiomyocytes (NMCMs, NRCMs) were isolated from 0-3 day old pups with the Neonatal Heart Dissociation Kit (Miltenyi) according to manufacturer’s instructions. Hearts were extracted and digested with in a gentleMACS C-tube and gentleMACS Octo Dissociator (Miltenyi). After pre-plating for minimum 90 min, cardiomyocytes were cultured in Minimum essential medium (Biocencept) supplemented with 5% FBS (Merck), 1% penicillin/streptomycin (Life Technologies), 100 nM BrdU (Sigma), 2 µg/ml vitamin B12 (Sigma) at 37 °C and 1% CO_2_.

Cardiomyocyte-like HL-1 cells were maintained in Claycomb medium (Sigma) supplemented with 10% FBS (Gibco), 0.1 mM norepinephrine (Sigma), 2 mM L-glutamine (Sigma), 1% penicillin/streptomycin (Life Technologies) at 37 °C and 5% CO_2_.

Human induced pluripotent stem cells (hiPSC) (MHHi001-A)^1^ were cultured in full StemMACS iPS-Brew XF medium (Miltenyi) on Geltrax-coated plates (Gibco) and passaged with Versene (Gibco) in medium supplemented with 2 µM Thiazovivin (Selleckchem). hiPSCs were subjected to cardiac differentiation by Wnt modulation with 5 µM GSK-3 inhibitor XVI (Millipore) for 48 h and 5 mM IWP-2 (Selleckchem) for 48 h in RPMI 1640 medium supplemented with human recombinant albumin (Sigma) and L-ascobic acid (Sigma) as described previously^2,3^. Metabolic selection for 4-10 days purified cardiomyocytes in 4 mM DL-lactate (Merck) in no glucose RPMI 1640 medium (Gibco) with albumin and L-ascobic acid. Cardiomyocytes were maintained in 1x B27 (Gibco) in RPMI 1640, GlutaMAX (Gibco) and applied for experiments at differentiation day 40-60.

HEK-293T cells were cultured according to standard protocols for AAV productions.

##### In vitro treatments and assays

Hypertrophic stimulation

Cardiomyocytes were treated with 5 nM Leukemia Inhibitory Factor (LIF, Merck), 100 µM phenylephrine/isoprenaline (PE/ISO, Sigma) or 5 nM cardiotrophin-1 (CT-1, Sigma) for 48 h.

Actinomyosin D

HL-1 cells were treated with 2 μg/ml actinomycin D (Sigma Aldrich) in Claycomb medium for 0.5 to 24 h.

RNase R treatment

3 μg RNA of HL-1 cells were treated with 3 Units RNase R (Biozym Scientific) in 1x RNase R buffer for 10 min at 37 °C, in parallel to a mock control without RNase R. 1 μg RNA was reverse transcribed.

WST-1

Cell viability was assessed using the Cell Proliferation Reagent WST-1 (Roche). NMCMs and hiPSC-CMs were cultured in 96-well plates. After adding 10 μl WST-1 reagent, plates were incubated at 37 °C for 2 h. Absorbance was measured at 450 nm and 630 nm using an HT Synergy plate reader (Biotek), providing a readout of cell viability based on metabolic activity.

*Seahorse XF Mito Stress Test*

Oxygen consumption rate (OCR) was determined in NRCMs via the Seahorse XF Mito Stress test (Agilent) and measured at the Seahorse XF96 Analyzer as described previously^2,3^. OCR was normalized to the cell count, determined by Hoechst (Thermo Fisher) staining.

*Measurement of intracellular Ca^2+^ transients with Fura-2 AM*

Intracellular Ca^2+^ transients in single cardiomyocytes were assessed with a dual excitation fluorescence photomultiplier system (IonOptix). hiPSC-CMs were incubated with 1.5 μM Fura-2 acetoxymethyl ester (AM) (Invitrogen) for 25-35 min at 37 °C and 5% CO_2_. Subsequently, cells were washed twice with medium for 15 min each. Fluorescence measurements were recorded from specific regions of interest (ROI) in individual cardiomyocytes. Emission at 510 nm, following alternating excitation at 340 and 380 nm, was captured using IonWizard software version 6.5 (IonOptix). Autofluorescence was subtracted using data from unloaded cardiomyocytes. For acute isoprenaline stimulation, 1 µM isoprenaline was infused to the perfusion chamber.

##### Modulation of circRYR2 expression

CircRNA knockdown was accomplished via RNA interference, utilizing siRNAs (Eurofins Genomics) specifically designed to target the backsplice site. As a negative control, scrambled siRNA was employed. Cardiomyocytes were transfected with 100 nM siRNA using Lipofectamine 2000 (Life Technologies) in Opti-MEM (Gibco). 4-8 h after transfection in NMCMs and HL-1 cells, medium was changed to their corresponding culture medium, while for hiPSC-CMs medium was changed after 12 h. Subsequent analyses were performed 48 h post-transfection.

To enable circRYR2 overexpression, a construct containing the circRYR2 sequence was designed and cloned into an AAV backbone. The overexpression plasmid contained exon 24 to exon 29 of the rodent RYR2 locus. As the circularization signal, upstream 100 bp of intron 23 and the upstream ALU element were included, downstream 100 bp of intron 29 and the downstream ALU element were employed. The plasmid was synthesized and incorporated into the pMA-RQ (AmpR) delivery vector (GeneArt® Gene Synthesis; Invitrogen). Same design was applied for the human overexpression construct.

Sequence of mmu-circRYR2 overexpression construct:

Green = sequence with restrictions sites for cloning

Blue = ALU elements

Red = circRYR2 sequence

Black = intronic regions

AGCAAGCTAGCGCAGTGGTACCCGGATCCAGTCCGTGGAATTCTTTTGGGGAAGCTTGGGAGCTCACTCAGTCTAAGGGTGCTAACTGCACCCACAGGAGGAATTTAGTTCAAATCCCCTGCATACACATAGGAAGTAGGCCTGACCACACACATCCACTGATAACTCCACAACTGCAGGAAGAGACAAAGACAAGAGGCTTGCTGACTACCAGGTAGCTCCCAGCTCAGTGAGAAACACTGTCTCAAGGGAATAACCAGGGCCAGCTGTCAGAGAGTTCTACCTCCTCATTTGTTCCCACACAGCTGTGTTCCACACTAGAAGCTCCTCTATACTCATTTCCCTTCCTGTTTCAGGTTAGAGATGACAACAAGAGACAGCACCCATGTCTGGTGGAGTTCTGCAAGCTTCCAGAACAGGAACGCAACTATAATTTGCAGATGTCGCTTGAAACCCTCAAGACTTTGCTGGCATTGGGATGTCACGTGGGTATAGCTGATGAACATGCTGAAGAGAAGGTGAAGAAAATGAAACTGCCTAAGAATTACCAGCTGACCAGCGGATACAAGCCTGCCCCAATGGATCTGAGCTTTATAAAACTAACCCCGTCTCAGGAAGCAATGGTGGACAAGTTGGCTGAAAATGCTCACAATGTGTGGGCCCGGGACCGAATCCGGCAAGGCTGGACTTACGGCATCCAGCAGGATGTAAAGAATAGAAGAAACCCTCGTCTCGTCCCCTACACTCTTCTGGATGACAGAACCAAGAAGTCCAACAAAGACAGTCTCCGAGAGGCAGTGCGCACACTGCTGGGCTACGGCTACCACCTGGAAGCTCCTGATCAGGACCATGCCTCGAGAGCCGAGGTGTGCAGCGGTACTGGGGAGAGGTTCCGCATCTTCAGGGCAGAGAAGACCTATGCGGTGAAGGCTGGGCGCTGGTACTTTGAATTTGAGGCTGTCACTGCTGGAGACATGCGGGTGGGCTGGAGCAGGCCAGGCTGCCAACCAGATCTAGAACTGGGTTCTGATGACCGTGCCTTTGCTTTTGATGGCTTTAAGGCACAGCGGTGGCACCAGGGCAATGAACACTACGGGCGCTCATGGCAAGCTGGTGACGTCGTGGGCTGCATGGTTGATATGAATGAACACACCATGATGTTCACTCTGAACGGCGAGATCCTGCTGGATGATTCAGGCTCTGAGCTGGCATTCAAGGACTTTGATGTTGGTGATGGTAAGAGCCACGGTGTTCTGTCATTTGGGTTTGCAGACAATGCATGGCCAGGCCCAATAGTAGTTTTAGGGTGAGTGGGTGTGTAAGTTGCCTCTGTAAAACCTCAGAGTTAGGAGGCTAAGGCAGGAGGATCCTGAGTTTGAGACCTGTCTGGGCTACCTAGTGAGTTCAAGGCTGGTCTGGGCTACGTAATGAACCTGGTAGGCATTAGCGGCCGCTCGAGTCCAAATTGCTGGGATTTAGAGGGCCCGTT

Sequence of hsa-circRYR2 overexpression construct:

AGCAAGCTAGCGCAGTGGTACCCGGATCCAGTCCGTGGAATTCTTTTGGGAGGAACATCTCCAGGGACTTCAAATAGCTACCTTACCATCTACTACTTGTAAGACCATAACCAAATTACTTAACCTCTCTTAATTTGGATGTCTTATAAACAGAACACCAAGTACCATCACCTTGTTTAGTTATTTTGACGATTAATTTGTCACTACTTATTCAAATCTTTTGACTTTGGCTCTGAAGCTGATTCTCTGAGATAAAAAAATACATGACCTTCCTTAATGTTTTCCCCCCAATAGAGGTTGTATTGCTCGTACTGTAAGCTCACCAAACCAACATCTGTTAAGAACTGATGATGTCATCAGTTGCTGTTTAGATCTGAGTGCCCCAAGCATCTCGTTCCGAATTAATGGACAACCTGTTCAAGGAATGTTTGAGAATTTCAACATCGATGGCCTCTTCTTTCCAGTCGTTAGTTTCTCTGCAGGAATAAAAGTACGCTTTCTGCTTGGAGGGCGACATGGAGAATTCAAATTTCTTCCTCCACCTGGGTATGCTCCTTGTTATGAAGCTGTTCTGCCAAAAGAAAAGTTGAAAGTGGAACACAGCCGAGAGTACAAGCAAGAAAGAACTTACACACGCGACCTGCTGGGCCCCACAGTTTCCCTGACGCAAGCTGCCTTCACACCCATCCCTGTGGATACCAGCCAGATCGTGTTGCCTCCTCATCTAGAAAGAATAAGAGAAAAACTGGCAGAGAATATCCATGAACTCTGGGTTATGAATAAAATTGAGCTTGGCTGGCAGTATGGTCCGGTTAGAGATGACAACAAGAGACAACACCCATGCCTGGTGGAGTTCTCCAAGCTGCCTGAACAGGAGCGCAATTACAACTTACAAATGTCGCTTGAGACCCTGAAGACTTTGTTGGCATTAGGATGTCATGTGGGTATATCAGATGAACATGCTGAAGACAAGGTGAAAAAAATGAAGCTACCCAAGAATTACCAGCTGACAAGTGGATACAAGCCTGCCCCTATGGACCTGAGCTTTATCAAACTCACCCCATCACAAGAAGCAATGGTGGACAAGTTGGCAGAAAATGCACATAATGTGTGGGCGCGGGATCGAATCCGGCAGGGCTGGACTTATGGCATCCAACAGGACGTAAAGAACAGAAGAAATCCTCGCCTTGTTCCCTACACTCTTCTGGATGACCGAACCAAGAAATCCAACAAGGACAGCCTCCGCGAGGCTGTGCGCACGCTGCTGGGGTACGGCTACAACTTGGAAGCACCAGATCAAGATCATGCAGCCAGAGCCGAAGTGTGCAGCGGCACCGGGGAAAGGTTCCGAATCTTCCGTGCCGAGAAGACCTATGCAGTGAAGGCCGGACGGTGGTATTTTGAATTTGAGACGGTCACTGCTGGAGACATGAGGGTTGGTTGGAGTCGTCCTGGTTGTCAACCGGATCAGGAGCTTGGCTCAGATGAACGTGCCTTTGCCTTTGATGGCTTCAAGGCCCAGCGGTGGCATCAGGGCAATGAACACTATGGGCGCTCTTGGCAAGCAGGCGATGTCGTGGGGTGTATGGTTGACATGAACGAACACACCATGATGTTCACACTGAATGGTGAAATCCTTCTTGATGATTCAGGCTCAGAACTGGCTTTCAAGGACTTTGATGTTGGCGATGGTCTGTAAGTCTACTATGTTTTGTGTTTTTTTTAAGTTTGCAGCACAAGGAAGCTTTCATCCTGAGGCTTCCTAACCGGGCGTTTCTGTTTCAGGGTGAGGGCCGGGTTCAGTGGCTCAAGCCTGTAATCCCAGCACTTTGGGAGGCCGAGGTGGATGGATCACAAGGTCAGGAGTTCGAGACCAGCCTGACCAACATAGTGAAACCCCATGTCTACTAAAAAATACAAAAAATTAGCTGGGCATGGTGGCATGCACCTGTAATCCCAGCTATTTGAGAGGCTGAGGCAGGAGAATTGCTCGAACCCAGGAGGCAGAGGTTGCAGTGAGCAGAGATCACGCCATGGCACTCCAGCCTGGGCAACAGTGCAAGACTGCATCTCAAAAAAAAAAAAAAAGTAATGAACCTGGTAGGCATTAgcggccgcTCGAGACGTGGGTAACCGTTATCCAAATTGCTGGGATTTAGAGGGCCCGTT

AAV production

Stbl3 *E. coli* were transformed with the overexpression vectors, empty AAV MCS 1.3 vector (negative control) or AAV helper plasmid PDP6rs were grown in LB medium with 100 μg/μl ampicillin. After 16 h of incubation at 37 °C shaking, cultures were expanded in LB medium with 100 μg/μl ampicillin and incubated overnight at 37 °C shaking. Plasmid DNA was extracted using the NucleoBond® Xtra Midi Kit (Macherey-Nagel) as per manufacturer's instructions. Plasmid sequences were confirmed by Sanger sequencing and restriction enzyme digestion. The PDP6rs plasmid was digested with ClaI and KpnI at 37 °C for 2 h, while the overexpression plasmids were digested with AhdI at 37 °C for 2 h and SmaI at 25 °C for 2 h. Digestion patterns were confirmed by agarose gel electrophoresis.

HEK293T cells were seeded at a density of 5 x 10^6^ cells per 15 cm culture dish in DMEM medium supplemented with 10% FBS and 1% P/S. Upon 80% confluency, cells were transfected with either AAV6 empty vector (AAV-MCS 1.3), AAV6 circRYR2 (mmu) or AAV6 hsa-circRYR2 constructs, along with pDP6rs and polyethylenimine HCl MAX, Linear (PEI MAX) (Supplementary Table 2).

Supplementary Table 2 Preparation of transfection mixture for AAV6 production

| For 10 dishes | Stock  concentration | AAV-empty  production | hsa/mmu-AAV6-circRYR2  production | Volume |
| --- | --- | --- | --- | --- |
| DMEM |  |  |  | 50 ml-(A+B+C) |
| AAV-construct-plasmid | 1 µg/µl | 100 μg (10 μg/plate) | 122 μg  (10 μg/plate) | (A) |
| AAV6-packaging-plasmid (pPDP6rs) | 1 µg/µl | 360 μg  (36 μg/plate) | 360 μg  (36 μg/plate) | (B) |
| PEI MAX | 1 mg/ml | 4x 460 μg  (1:4 ratio)  = 1840 μg | 4x 482 μg  (1:4 ratio)  = 1928 μg | (C) |
| Total |  |  |  | 50 ml |

Cardiomyocytes were transduced for 72 h with AAV6 empty or AAV6 circRYR2 virus at a 1x10^4^ multiplicity of infection (MOI).

##### Gene expression analysis

RNA was isolated from cultured cells using with TriFast, Qiazol or by the miRNeasy Kit according to manufacturer guidelines. Briefly, cells were lysed in TriFast or Qiazol and incubated at room temperature for 5 min and mixed with 140 µl chloroform, vortexed and centrifuged for 20 min at 12000 xg and 4 °C. The aqueous phase was mixed with an equal volume of isopropanol and centrifuged for 10 min. The RNA pellet was washed twice with 75% ethanol, air-dried, and dissolved in nuclease-free water. When RNA was isolated with the miRNeasy Kit, cells were lysed in 700 µl Qiazol, followed by chloroform addition and centrifugation. The aqueous phase was applied to the spin columns, optionally treated with DNase and eluted in nuclease-free water. RNA quality and quantity were measured using a Synergy HT Reader (BioTek) at 260/280 nm.

cDNA was synthesized using the Biozym cDNA Synthesis Kit following the manufacturer’s instructions. Initially RNA (500-1000 ng) was treated with DNase I (Qiagen) to remove gDNA, then reverse transcribed with hexamer primers. A -RT control was performed to ensure the absence of DNA contamination. The cDNA synthesis was performed using a T3000 thermocycler (Biometra), with an initial incubation at 30 °C for 10 min, 60 min at 50 °C, and enzyme inactivation at 99 °C for 5 min. The cDNA was diluted to a 10 ng/μl. RT-qPCR was with iQ SYBR® Green Super Mix (Bio-Rad), ROX reference dye (Thermo Fisher), Precision Blue Real Time PCR Dye (Bio-Rad), and a primer mix (Supplementary Table 3) on a QuantStudio or ViiA7 Real-Time PCR systems. Each well received 2 µl of cDNA (20 ng). Data were analysed by ΔΔCt, normalizing target gene Ct values to housekeeping gene and controls.

Supplementary Table 3 Primers used for qPCR, genotyping and plasmid cloning in the study

| **Murine qPCR primers** | | |
| --- | --- | --- |
| **Gene** | **Forward** | **Reverse** |
| *Hprt* | GCGTCGTGATTAGCGATGAT | TCCTTCATGACATCTCGAGCA |
| *Tbp* | CCACTCACAGACTCTCACAAC | CTGCGGTACAATCCCAGAACT |
| *18S* | GTAACCCGTTGAACCCCATT | CCATCCAATCGGTAGTAGCG |
| circRYR2 | CGAGATCCTGCTGGATGATT | GTTTCAAGCGACATCTGCAA |
| linearRYR2 | CTAATGTCTGGGTGGGCTGG | TGCTGCGTTTGATGCTCTCA |
| pre-circRYR2 | GCCAGCTGTCAGAGAGTTCT | CATCCCAATGCCAGCAAAGT |
| circHIPK3 | GGATCGGCCAGTCATGTATC | ACCGCTTGGCTCTACTTTGA |
| circN4BP2L | GTTACCGGATGACCCAACAC | CAGGCGCAAGGTTACCTAAG |
| circPHC3 | GCTTCGGGATGTGAGGATTA | TTGGTGAGCCATTCTGTGAG |
| circICA1 | GCATGGAGCAGTACAGGACA | CCTGCAACTCCCAGGAATAA |
| *Actb* | ATCAAGATCATTGCTCCTCCTG | AGGGTGTAAAACGCAGCTCA |
| *Atp2b4* | TCCACGGTGAGAAGAACGTG | CTGATTCTCACTCACTGCCCA |
| *pCMV* | GCGTGGATAGCGGTTTGACT | GGGCGGAGTTGTTACGACAT |
| *Nppb* | CTGAAGGTGCTGTCCCAGAT | GTTCTTTTGTGAGGCCTTGG |
| *Nppa* | GCTTCGGGGGTAGGATTGAC | AGACCCCACTAGACCACTCA |
| *Rcan1* | CTGCACAAGACCGAGTT | TGTTTGTCGGGATTGG |
| *C-fos* | CAGCTCCCACCAGTGTCTAC | TGGCACTAGAGACGGACAGA |
| *Serca2a* | TCAGTATGACGGGCTTGTAG | CGGTAGCTTCTCCAACTTTC |
| *Pln* | GACGATCACCGAAGCCAA | CGAGCGAGTGAGGTATTGC |
| **Human qPCR primers** | | |
| **Gene** | **Forward** | **Reverse** |
| *HPRT* | AGGACTGAACGTCTTGCTCG | GTCCCCTGTTGACTGGTCATT |
| circRYR2 | GTTGGCGATGGTTGTATTGC | TGAATTCTCCATGTCGCCCT |
| LinearRYR2 | GTCTGGGTGGGCTGGATTAC | GCTGCGTTTGATGCTTTCAT |
| Pre-circRYR2 | TTGACATGAACGAACACACCA | TCATCCTACCCACTTGAGCC |
| *NPPB* | CAAGATGGTGCAAGGGTCTG | TTCCTCTTAATGCCGCCTCA |
| *NPPA* | ACTCCTCTGATCGATCTGCC | CCTCCCTGGCTGTTATCTTCA |
| *RCAN1* | GTGCCGGGCCAAATTTGAG | GCCAGGTGTGAGCTTCCTAT |
| *Cav1.2* | ACTGGTCAGGGATGATTGGG | AGTAACTATGGCCCGAGACG |
| *CSQ* | CCTCTACTACCATGAGCCGG | TGCTGATGATCTCCACTGGG |
| *PLN* | GATGATCACAGCTGCCAAGG | AGCTGAGCGAGTGAGGTATT |
| *SERCA2a* | AGAGAACACCCCTTCAGCAA | GAGGCTTCGAACAATGGCAT |
| *ATP2B4* | GCAATACCTACCCGATCCCTG | ACCGCATTGTTGTTTGTATTGG |
| *pCMV* | GCGTGGATAGCGGTTTGACT | GGGCGGAGTTGTTACGACAT |
| **siRNA** | **Sense Strand Sequence 5' -> 3'** |  |
| siRNA control | AGGUAGUGUAAUCGCCUUG**(**dTdT**)** | |
| mmu-siRNA circRYR2 | GGUGAUGGUUAGAGAUGACAA(dTdT) |  |
| hsa-siRNA circRYR2 | AUGUUGGCGAUGGUUGUAUUG(dTdT) | |

##### Genomic DNA Isolation

Genomic DNA (gDNA) was isolated using the DNeasy Blood & Tissue Kit (Qiagen) following manufacturer’s instructions. The isolated gDNA was diluted in 200-400 µl nuclease-free water and concentration was assessed using a Synergy HT Reader (BioTek Instruments Inc.) at 260/280 nm.

##### Polymerase chain reaction

PCR was conducted using the HotStarTaq Master Mix Kit (Qiagen). A 10 μl reaction mix included 0.5 μl each of forward and reverse primers (10 μM), 5 μl master mix, and 1-200 ng template DNA, adjusted with nuclease-free water. 35-40 cycles of 94 °C for 1 min, 30 s at 60 °C and 72 °C for 1 min were performed with an initial step of 95 °C 15 min and a final step of 72 °C for 10 min.

##### Agarose gel electrophoresis

Gel electrophoresis was conducted to visualize and isolate DNA fragments. DNA samples were prepared with 1x loading dye. Agarose was dissolved in 1x TAE buffer and Midori Green (Biozym). Samples and Quick-Load DNA ladder (NEB) were loaded on the polymerized gel. The gel was imaged using an INTAS Gel Doc system. Specific DNA fragments needed for further experiments were excised from the gel.

##### Gel extraction and Sanger sequencing

DNA was extracted from agarose gels using the QIAquick Gel Extraction Kit, following the manufacturer's instructions. Briefly, DNA bands were excised and weighed. Three volumes of QG buffer were added per volume of gel, and the mixture was incubated at 50 °C for 10 min with vortexing. An equal volume of isopropanol was added. The solution was transferred to a column containing a silica gel membrane and centrifuged at 13000 rpm for 1 min. The membrane was washed with Buffer PE, incubated for 2-5 min, and centrifuged again. DNA was eluted with nucleic acid-free water and concentration was measured by absorbance at 260 and 280 nm using Synergy HT (BioTek) with the Take3 Plate and Gen5 software.

For DNA sequencing a premixed reaction containing 15 μl DNA (2 ng/μl) and 2 μl of a specific primer (10 μM) were sent to Eurofins MWG Operon.

##### Western Blotting

Proteins were isolated from cells in 1x Cell Lysis Buffer (Cell Signalling Technology) with 1 mg/ml Pefabloc SC AEBSF (Sigma) by vortexing and on ice. After centrifugation at 8000 xg and 4 °C, protein concentration was determined with a Bradford assay. Samples were diluted and mixed with 1x Roti-Quant (Roth) 1:5. Absorbance was measured at 595 nm at a Synergy HT8 (BioTek) and determined via a standard curve.

For the SDS-PAGE, gels were prepared in the required SDS concentration. Samples were incubated at 95 °C for 5 min supplemented with 1x Loading buffer and DTT (NEB) and loaded onto the gel. After separation, proteins were transferred to a PVDF membrane (Bio-Rad). The membrane was activated in ethanol for 1 min and rinsed in water for 2 min and incubated in 1x transfer buffer for 5-10 min. Blotting was performed at 100 V for 90 min or 30 V overnight. Membranes were blocked in 5% milk in 1x TBST for 1 h. After incubation with the primary antibody in 5% milk in TBST overnight at 4 °C (Supplementary Table 4), membranes were washed three times in TBST. After 1 h incubation with the HRP-conjugated secondary antibody and Precision StrepTactin-HRP (1:10000, Bio-Rad), bands were visualised after washing with Clarity Western ECL Substrate or Clarity Max Western ECL Substrate (Bio-Rad) at a ChemiDoc system (Bio-Rad). Protein levels were normalized to GAPDH as a house keeper or for p-PLN to total PLN.

Supplementary Table 4 Antibodies applied for Western blotting

| **Antigen** | **Supplier** | **Dilution** |
| --- | --- | --- |
| CSQ | 26665-1-AP, Proteintech | 1:3000 |
| GAPDH | ab8245, Abcam | 1:10000 |
| Cav1.2 | ab81980, Abcam | 1:3000 |
| NCX1 | ab6495, Abcam | 1:1000 |
| RYR2 | 19765-1-AP, Proteintech | 1:1000 |
| SERCA2 | ab2817, Abcam | 1:1000 |
| PLN | #A010-14, Badrilla | 1:5000 |
| p-PLN (p-Ser16) | #A010-12AP, Badrilla | 1:5000 |
| anti-goat | ab7132, Abcam | 1:10000 |
| anti-mouse | #7076, Cell Signaling | 1:10000 |
| anti-rabbit | #7074, Cell Signaling | 1:10000 |

##### RNA sequencing

Whole transcriptome analysis was performed on total RNA was extracted from the hearts of four mice 3 weeks after TAC or sham surgery. Ribosomal RNA was depleted, RNA integrity was assessed with a Bioanalyzer (Agilent). RNA sequencing libraries were prepared with the Illumina TruSeq kit according to manufacturer’s instructions and sequenced on an Illumina HiSeq 2000 system, generating 100 bp paired-end reads. Reads were aligned to the circBase database (mm10, Ensembl) to identify circRNAs using Bowtie2 in sensitive mode, followed by feature counting to obtain raw read counts. For RNA sequencing in NMCMs, total RNA was isolated after circRYR2 modulation using the miRNeasy kit. The mRNA enrichment was performed using the NEBNext® Poly (A) mRNA Magnetic Isolation Module (NEB). Stranded cDNA libraries were prepared with the NEBNext® Ultra Directional RNA Library Prep Kit for Illumina. These libraries were sequenced on an Illumina HiSeq550 with a 2x75 bp paired-end read configuration at a read depth of 30 million per sample. The sequencing data were aligned to the mouse reference genome (mm10, Ensembl) using the STAR aligner in default mode and feature counts were used to obtain raw read counts.

##### Immunostaining

NMCMs were seeded in 96-well plates at a density of 15000 cells per well. After treatment, cells were fixed with 4% paraformaldehyde (Roth) for 10 min and permeabilized with 0.1% Triton X-100 (Roth) for 10 min. After blocking in 5% donkey serum (Serotec) in PBS for 30 min, cells were incubated with antibody against cardiac troponin T (cTnT) antibody (1:1000, Abcam, ab8295) at 4 °C overnight. The next day, cells were incubated for 30 min with anti-mouse Alexa Fluor 594 secondary antibody (1:500, Invitrogen) and Hoechst dye (1:10000, Thermo Fisher). Imaging was conducted at the Nikon Eclipse Ti microscope and analysis was performed with NIS Elements software.

**Supplementary References**

1. Haase, A., Gohring, G. & Martin, U. Generation of non-transgenic iPS cells from human cord blood CD34(+) cells under animal component-free conditions. *Stem Cell Res* 21, 71–73 (2017).

2. Lu, D. *et al.* A circular RNA derived from the insulin receptor locus protects against doxorubicin-induced cardiotoxicity. *Eur Heart J* 43, 4496–4511 (2022).

3. Neufeldt, D. *et al.* Circular RNA circZFPM2 regulates cardiomyocyte hypertrophy and survival. *Basic Res Cardiol* 1–20 (2024) doi:10.1007/s00395-024-01048-y.
